# Development and initial implementation of the Dynamic Assessment Treatment Algorithm (DATA)

**DOI:** 10.1371/journal.pone.0178806

**Published:** 2017-06-27

**Authors:** Katya C. Fernandez, Aaron J. Fisher, Cyrus Chi

**Affiliations:** Department of Psychology, University of California, Berkeley, Berkeley, California, United States of America; University of Amsterdam, NETHERLANDS

## Abstract

Given the recent increase in transdiagnostic research, it is important to discern how dimensional models of psychopathology could be used to guide personalized, dynamic assessment and treatment of symptoms. Using the person-specific approach described by Fisher (2015), we devised an initial 4-step algorithm for devising a treatment plan based on modular cognitive behavioral therapy using results obtained from within-person factor analyses and dynamic factor models. Then, we describe the improvement and digitization of the algorithm, termed Dynamic Assessment Treatment Algorithm (DATA). The development, structure, and clinical implications of DATA are discussed.

## Introduction

For decades researchers have sought to identify dimensional, transdiagnostic factors that account for the pronounced covariation found among psychological disorders in the Diagnostic and Statistical Manual of Mental Disorders (DSM) [1]. Among the mood and anxiety disorders, models such as Clark and Watson’s Tripartite Model [2] and its elaboration, the Integrative Hierarchical Model [3, 4], posited that much of the shared variance in these DSM-defined disorders stemmed from an underlying source of negative affectivity (NA). More recently there has been an increase in transdiagnostic research and ─ with the advent of the National Institute of Mental Health (NIMH)’s Research Domain Criteria Project (RDoC) ─ a structural shift toward investigating dimensional models of psychopathology. With the RDoC, the NIMH has indicated an interest in moving away from the relatively reliable, but potentially less valid categorical nosology of the DSM, to a more ostensibly valid dimensional system. Several dimensions among the mood and anxiety disorders have been identified, including negative and positive affect [4], neuroticism and extraversion [5], experiential avoidance [6], and sleep disturbances [7]. However, the process of mapping current mood and anxiety symptoms, as defined by the DSM-5 [1], onto a transdiagnostic framework—and how such a framework could be translated to a practical, user-friendly format for clinicians in the community—remains unclear.

The NIMH [8], its parent organization the National Institutes of Health, as well as the Food and Drug Administration have all recently emphasized the need for personalized medicine [9]–systems of assessment and intervention that utilize precision diagnostics to provide targeted treatments. Broadly speaking, personalized medicine works under the assumption that more precise delineations of individual needs will lead to increasingly more effective interventions. In medicine, researchers employ molecular genetic methodologies to attempt to discern genetic profiles that confer increased risks or benefits from a given treatment [9]. Whereas some investigators are pursuing similar methods for better understanding psychosocial interventions [10], it has been argued that the required sample sizes are far too large for molecular genetics to exclusively bear the burden of personalization targets in psychosocial treatment. As the field shifts towards dimensional, transdiagnostic models of psychopathology, it stands to reason that dimensional modeling of individual symptomatology could potentiate new, possibly finer-grained treatment targets–compared to the relatively macroscopic and polythetic diagnostic categories of the DSM. However, the exact mechanism whereby such a transdiagnostic system could be utilized in the context of personalized treatment remains unclear.

### From diagnoses to dimensions

Recently, Fisher [11] presented a dynamic model of psychological assessment capable of determining the structure of psychopathological syndromes on a person-by-person basis. This model does not presuppose a categorical diagnosis or a hierarchy of symptom predominance. Instead, the proposed model utilizes a bottom-up, data-driven approach to psychopathology that utilizes the covariation in symptoms and behaviors in time in order to identify latent, symptom-related dimensions on a person-by-person basis. This approach replaces standard nomothetic (i.e., between-subjects) analyses requiring adequate numbers of *participants* with time series methodologies that require an equivalent number of *observations* within each individual. Although traditional nomothetic research has been instrumental in beginning to identify the transdiagnostic dimensions underlying psychiatric disorders in the population, these data may not generalize to individuals. That is, understanding the factors that best explain the covariation among psychiatric symptoms between subjects does not necessarily provide information about the dynamics within and between constructs within a single individual; for a detailed explanation, see [12].

In a sample of individuals with generalized anxiety disorder (GAD), Fisher [11] found that a person-specific, dynamic assessment approach yielded fine-grained, dimensional information about psychopathology within individuals that would otherwise have been lost if the primary goal of assessment was to determine the absence or presence of a given diagnosis. For example, though all of the individuals in this study met criteria for GAD, the number and nature of latent factors that emerged from person-specific analysis differed across individuals (including dimensions for worry, avoidance, and fatigue). Moreover, dynamic analyses of the interrelationships between factors revealed actionable information about which dimensions predicted others in time. Such information about presence and primacy of symptoms is lost in assessment approaches focused on diagnostic categories, ignoring the underlying mechanics of the syndrome components.

### Utility of a person-specific approach

As we outline below, the delineation of person-specific syndrome structures and inter-symptom dynamics provides actionable information about each individual that facilitates the implementation of personalized interventions fine-tuned to individual needs. As Fisher [11] has argued, personalized treatment research requires data that accurately reflect individual processes. This requires data collected at intensive repeated intervals, as well as a set of specialized methodologies which include–but are by no means limited to–P-technique factor analysis [13] and dynamic factor modeling [14]. To date, there have been several attempts to conduct personalized assessment of psychological symptoms. For example, Van Der Krieke and colleagues [15] conducted a study in which participants completed questionnaires about themselves three times per day for 30 days and then provided participants with feedback that included descriptive information about their symptoms and network models (maximum of six symptoms) of how their symptoms related to each other. These authors captured symptom dynamics via network analysis, but they did not (a) directly assess symptom structures and (b) propose a way to connect descriptive information about symptoms and symptom dynamics to treatment. Though there are several research studies attempting to personalize treatment, these studies largely refer to personalization at the treatment type selection level (e.g., whether a patient will receive cognitive behavioral therapy or psychotropic medication [16]).

To better understand how symptoms are structured within an individual, we focused on the use of person-specific factor analysis, often referred to as P-technique. Although underutilized in current psychiatric and psychological research, the factor analysis of person-level data was first employed by Cattell [13] and has been discussed in detail by recent scholars such Molenaar [12] and Nesselroade [17]. Fisher demonstrated that P-technique can be successfully applied to intensive repeated measures of DSM-related symptoms to return idiosyncratic factor structures on a person-by-person basis. Analyzing twelve such symptoms (including two dimensions of worry, the five associated DSM-5 symptom criteria for GAD, and five related avoidance behaviors), Fisher found that individual models of generalized anxiety and avoidance could be represented with 3 to 4 latent dimensions. The most prevalent dimension was related to avoidance behaviors (90% of sample), with worry- and fatigue-related dimensions being the second most prevalent (70% of sample each). Moreover, the dynamic assessment model provided information about the strength of association between each symptom and factor. In fact, standardized factor loadings provide an easily interpretable metric for understanding the contribution of each symptom to the overall syndrome structure.

**Fig 1:**
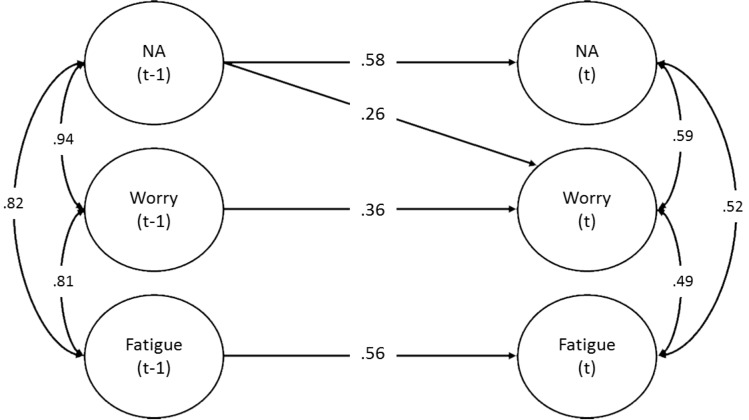
Dynamic factor model for participant 021. NA = negative affect; (t-1) = observation of one lag; (t) = observation in aggregate current time. The time lag for this model is approximately 6 hours.

Importantly, the dynamic assessment model goes beyond the identification of latent dimensions, further elaborating the individual nature of psychopathology by articulating the temporally-structured relationships among these dimensions in time. That is, after determining the composition (i.e. structure) of individual syndromes, dynamic analyses assess the contemporaneous correlations and time-lagged predictive relationships within and between dimensions in time, allowing investigators to directly assess the temporal stability of each dimension, as well as the degree to which one dimension drives another in time. In this way the researcher can directly assess the potential influence of one symptom domain on another–information that can be used to inform the selection and preferential ordering of modules in a personalized, modular intervention. Fig 1 presents a three-dimensioned dynamic factor model with a lag of one observation for a participant in the pilot study described below. This model depicts the relationships among dimensions from one observation to the next (approximately 6 hours). For example, in this figure, NA at one time point moderately predicted NA at the next time point (.58) (i.e., NA at one time point predicted 34% of the variance in NA levels approximately 6 hours later). NA also significantly (.26), though less-strongly, predicted worry from one time point to the next (i.e., NA at one time point predicted 7% of the variance in worry levels approximately 6 hours later). Thus, dynamic factor models allow for relationships among the dimensions over time to be explored.

### Modular psychotherapy

Once the symptom dynamics are identified and articulated for each individual, the next step is to create a method whereby such factor structures are directly incorporated into treatment planning. A natural avenue of exploration is a modular psychotherapy that allows for the selection of specific modules based on presenting symptom structures and dynamics. For example, individuals whose factor structures reveal cognitive symptoms (such as worry, rumination, and catastrophic thinking) will likely benefit from the delivery of a streamlined cognitive reappraisal module, whereas individuals presenting with a more variegated negative affect factor will likely benefit from a correspondingly broader approach including psychoeducation and emotion awareness, in addition to reappraisal strategies. Cognitive behavioral therapy (CBT) is an excellent starting place for creating modularized treatments for mood and anxiety symptoms, given the abundance of empirical support for CBT treatments of mood and anxiety disorders [18]. Moreover, the symptom-based structure of CBT makes it easily compartmentalized into component interventions such as psychoeducation, reappraisal, exposure, applied relaxation, mindfulness, and so forth. The present study used the Unified Protocol for Transdiagnostic Treatment of Emotional Disorders (UP) [19], given its added strength as a single treatment for all mood and anxiety disorders and explicitly modular design. The UP is designed to treat emotional disorders from a transdiagnostic perspective rather than to focus on specific clusters of symptoms comprising diagnostic categories. The UP is composed of eight modules: motivation enhancement for treatment engagement (Module 1), psychoeducation and tracking of emotional experiences (Module 2), emotion awareness training (Module 3), cognitive appraisal and reappraisal (Module 4), emotion avoidance and emotion-driven behaviors (Module 5), awareness and tolerance of physical sensations (Module 6), interoceptive and situation-based emotion exposures (Module 7), and relapse prevention (Module 8). It is worth noting that although the authors of the UP refer to it as a modular therapy, it is routinely delivered in sequence, in its entirety.

### Translating person-specific assessment approaches to modular CBT: A pilot study

Following the recommendations of Fisher [11], our team initiated a personalized treatment study that utilized intensive repeated measures data to generate targeted, modular interventions for individuals with a primary diagnosis of major depressive disorder (MDD), GAD, or both. All participants completed an initial diagnostic interview, the Anxiety Disorders Interview Schedule, [20] and then completed surveys on their phone (received as a text messages, hyperlinked to web-based surveys) four-times-per-day for at least 30 days. The IRB at the University of California, Berkeley approved this study. Written consent was obtained, and participants were given the opportunity to ask questions prior to signing the consent form. Consent forms were stored in a secure, locked filing cabinet in a locked laboratory.

#### Measures

Phone surveys contained 26 items related to affect, avoidance behavior, and DSM symptom criteria for GAD and MDD (see S1 File for all items). Participants were asked to rate their experience of each symptom since the previous survey on a 0 (*not at all*) to 100 (*as much as possible*) visual analog slider, and were required to complete at least 80% of phone surveys to provide a sufficient sample size for factor analysis and dynamic factor modeling (~100 to 120 observations). If a participant’s completion rate dropped below 80% (the threshold for inclusion in the therapy trial), a reminder call was made to participants. Each multivariate time series obtained from the data was subjected to a person-specific factor analysis to identify latent pathological dimensions across symptoms of anxiety and depression. A 13-item subset of the complete survey was utilized for these analyses. These items included all DSM diagnostic criteria for GAD and MDD except suicidality, weight gain/loss, and sleep disturbance–with the former symptom omitted for safety concerns. Sleep, measurable only once-per-day, was omitted as analyses contained items measured four-times-per-day; and changes in weight were not considered fast-moving enough to exhibit meaningful variation over the measurement period. In addition, three avoidance behaviors (reassurance seeking, procrastination, and avoiding activities) were included in our analyses.

#### Data analytic strategy

Given that the DSM-5 currently eschews avoidance behaviors for GAD and MDD, we took a two-step approach to incorporating the three avoidance items. Following the iterative approach outlined in Fisher [11], we fit two separate factor models–one without and then one with avoidance items included. If the avoidance items increased the percent of variance predicted without degrading model fit, these items were retained in the final model. Global model fit was evaluated using the: (a) Tucker-Lewis incremental fit index (TLI) [21], (b) comparative fit index (CFI) [22], and (c) standardized root mean square residual (SRMR) [23]. All indices were evaluated with the aid of recommendations by Hu and Bentler [24]. The returned factors were then used to factor-score the original time series and conduct a single-indicator dynamic factor analysis [14]. After data were analyzed, our treatment team–consisting of the principal investigator, two postdoctoral clinical psychologists, and a licensed, practicing clinical psychologist–met to review the collective results of each analysis.

### A two-stage approach to constructing an automated algorithm

Our team sought to construct a quantifiable and repeatable treatment-selection system that translates clinical decision-making into a set of digitized, *a priori* decision rules about the relationship between symptom presentation and appropriate intervention. Given the novelty of this paradigm and the real-world consequences for patients, we chose not to use a data simulation approach to constructing an algorithm. Instead, we chose to undertake a two-stage approach, in which we first codified our data collection and analysis procedures and approached the treatment-selection process within a relatively traditional functional-analytic methodology. That is, we assessed the presence and relative influence of various symptoms and behaviors, as they were reflected in the data analyses (see below for greater detail). Then, after an appreciable number of complete cases we piloted the digitization of an automated algorithm using these data. As described below, this was undertaken with 20 patients with acceptable pre-treatment dynamic assessment data, of which 14 were treatment completers.

## Stage 1: Treatment team decision model

The treatment team weighed the relative contribution of items to each factor and applied labels such as “Negative Affect,” “Depression,” “Anxiety,” and “Avoidance,” to the latent dimensions. The dynamic models were inspected for the strength of the autoregressions (i.e., the relative persistence or stability of each factor) and for the presence of cross-predictions. The latter represents a time-lagged predictive effect of one factor on another. Thus, in the presence of a cross-predictive parameter it can be inferred that therapeutic progress in the predictive factor at time (t-1) will have downstream effects on the dependent factor at time (t). Emphasis was therefore given to factors shown to drive the variation in other factors. Modules were selected and ordered based on their perceived fit to the most impactful factors–those factors which accounted for the greatest amount of variance within P-technique factor analyses and those with strong time-lagged effects (of either an autoregressive or cross-predictive nature).

**Fig 2:**
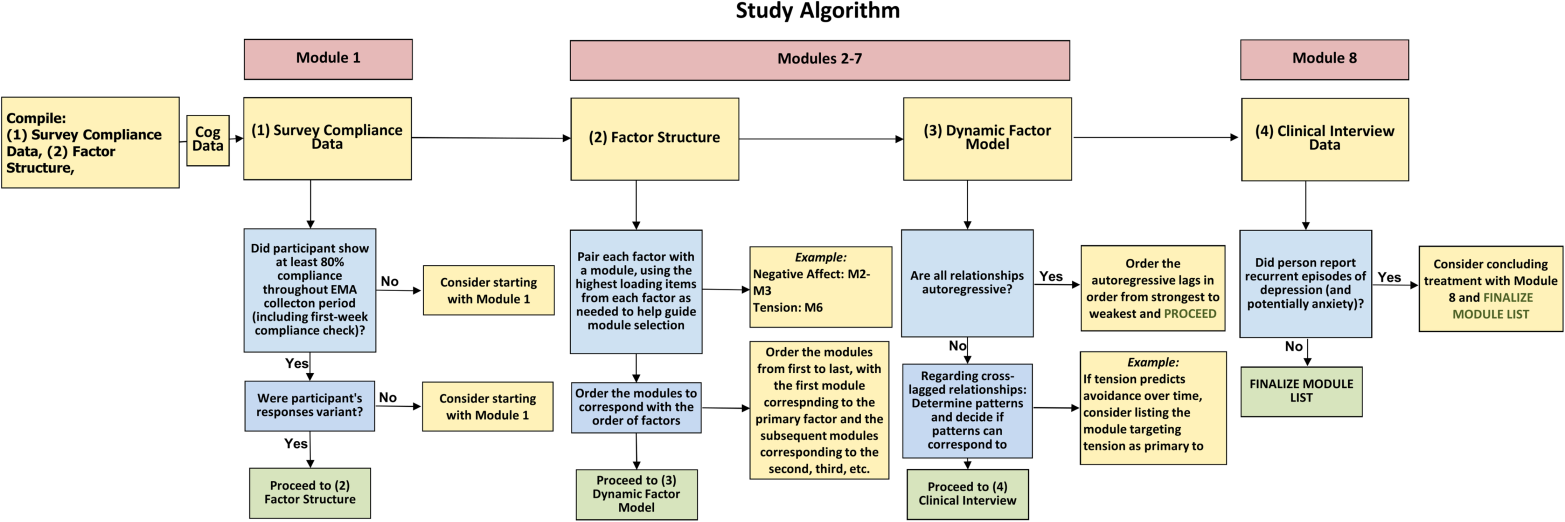
Treatment team decision model.

We codified this procedure into the 4-step selection algorithm presented in Fig 2. Step 1 utilized survey compliance data to discern whether the motivation enhancement module of the UP (Module 1) would be helpful to include in the treatment plan. The motivation enhancement module was incorporated into the treatment plan for participants who received reminder calls due to low completion rate for the phone surveys, or for any participants who needed more than 30 days to reach the required 80% completion rate. Step 2 utilized the factor structure data to determine which modules would be most helpful in addressing specific mood and anxiety symptoms. There were no *a priori* factor-module matches; rather, the treatment team examined each factor individually to determine the core construct underlying each factor and then agreed on an appropriate module to address that factor. For example, a factor comprised of cognitive symptoms (e.g., *worrying*, *feeling worthless/guilty*) was matched with the cognitive appraisal and reappraisal module.

Step 3 utilized the dynamic factor model to help determine the predominance of symptom dimensions and thus the preferential ordering of selected modules. If any cross-lagged relationships emerged, those relationships were considered when deciding the ordering of the modules. For example, if cognitive symptoms were driving negative emotionality, the module focusing on cognitive symptoms would precede the modules focusing on negative emotionality, based on the underlying assumption that positive changes in the temporally-predominant factor would have downstream effects on the temporally-dependent factor. Finally, Step 4 utilized GAD and MDD recurrence data obtained during the clinical interview to determine whether the relapse prevention module (Module 8) would be included in the treatment plan; that is, if the participant’s diagnosis was recurrent, the relapse prevention module was included in the treatment plan as the final module.

This treatment-selection procedure was applied to the first 20 patients eligible for treatment, 14 of whom went on to complete therapy; these data will be published in a primary outcome paper following the completion of the study. With these data in hand, we sought to further codify our treatment-selection process. Specifically, we sought to better operationalize the respective weights of the within-time variance accounted for (via the P-technique analysis) versus the degree of between-time predictive variance accounted for (via the dynamic analysis). Moreover, the semantically-oriented approach of relying on factor labels to operationalize the contents of each factor was revealed to be an insufficient metric of item contributions. For example, a latent dimension containing primarily cognitive symptoms would at times also contain symptoms traditionally associated with negative affect or avoidance. Thus, we sought to design an automated selection algorithm that considered the total contribution of each individual’s data: the strength and nature of contemporaneous covariance as reflected in the P-technique analysis, the nature of the time-lagged relationships–including both autoregressions and cross-predictions–in the dynamic analyses, and the mean levels of symptom severity as reported by each respondent. The resulting algorithm, termed Dynamic Assessment Treatment Algorithm (DATA), uses a series of equations to (a) quantify the strength of each factor based on its predictive power within time and across time via a factor score, (b) utilize standardized factor loadings to quantify the strength of association between the factor scores and each item, (c) weight the item/factor relationship by mean level (i.e. symptom severity) of each item, (d) and map items onto specific interventions via an item-module matching matrix.

## Stage 2: The Dynamic Assessment Treatment Algorithm

The following details the construction of DATA. Here we explain how the contents of the factor analyses and dynamic models–in concert with the average item level–are used in the calculation of an item score for each item and how the relationships within the item-module matching matrix dictate the selection and ordering of modules for personalized treatment construction. It should be noted that DATA focuses on Modules 2–7; Modules 1 and 8 were still assigned using the steps delineated in Fig 2.

### Selection of the item-module matching matrix

The foundation of DATA is built from the input items (i.e., symptoms and behaviors) and their relationship with specified, targeted interventions. The use of a semantic framework for determining the relative contribution of each factor to the presenting pathology can potentially undervalue the incremental contribution of individual items relative to the semantic categorization of the factor. Table 1 provides an example of this phenomenon. Participant 021 exhibited a three-factor solution, for which the three avoidance items degraded fit and were excluded. Note that the three factors, termed NA, worry, and fatigue, accounted for 30%, 24%, and 22% of the overall variance respectively. As depicted in Fig 1, the NA symptoms appear to drive variation in worry from observation to observation. Thus, using the Stage 1 version of the algorithm, we provided interventions aimed at targeting negative emotions (Modules 2 and 3), followed by a reappraisal intervention for worry and negative cognition (Module 4). Finally, an intervention for tolerating physical sensations (Module 6) was employed for fatigue and associated muscle tension. However, no interventions were provided for anhedonia–the loss of interest or pleasure in daily activities. Although the second-lowest loading, anhedonia nevertheless exhibited a .42 loading on the ostensibly most important factor (i.e., the factor accounting for the greatest percentage of variance, and the only factor demonstrating cross-predictive effects in time). We thus constructed a system wherein items would be awarded scores based on the strength of the factors they are associated with and the strength of relationship between the item and the factor (i.e., the factor loading). Module selection would then result from the rank-ordered importance of the items related to a given module. However, this required the codification of the item-module relationships. We therefore constructed a logical matrix (i.e., binary matrix) matching modules to constituent items. This item-module matching matrix was created by matching presenting symptoms and behaviors with relevant interventions, based on empirically-supported cognitive-behavioral treatments. The item-module matching matrix employed in the current study is presented in Table 2.

**Table 1:** P-technique results for participant 021.

	Factor Name (% of Total Variance Accounted For)		
** **	NA (30%)	Worry (24%)	Fatigue (22%)
**Irritable**	.91		
**Restless**	.35		.52
**Worried**		.97	
**Worthless/Guilty**	.49	.49	
**Loss of interest or pleasure**	.42		.47
**Hopeless**	.55	.34	
**Down/depressed**	.50	.38	
**Fatigue**			.78
**Muscle Tension**			.61

*Note*: NA = negative affect.

**Table 2:** DATA item-module matching matrix.

**Item**	**Module 2**	**Module 3**	**Module 4**	**Module 5**	**Module 6**	**Module 7**
**Felt irritable**	1	1	0	0	0	0
**Felt restless**	0	1	0	0	0	0
**Felt worried**	0	0	1	0	0	0
**Felt worthless or guilty**	1	0	1	0	0	0
**Experienced loss of interest or pleasure**	1	0	0	1	0	0
**Felt hopeless**	1	1	1	0	0	0
**Felt down or depressed**	1	1	0	0	0	0
**Felt fatigued**	0	0	0	0	1	0
**Experienced muscle tension**	0	0	0	0	1	0
**Had difficulty concentrating**	0	1	0	0	0	0
**Avoided activities**	0	0	0	1	0	1
**Sought reassurance**	0	0	0	1	0	1
**Procrastinated**	0	0	0	1	0	1

*Note*. DATA = Dynamic Assessment Treatment Algorithm. Items are from the phone survey administered four times per day for 30 days. Modules refer to modules from the *Unified Protocol for the Transdiagnostic Treatment of Emotional Disorders[25].*

### DATA structure

The following seven equations constitute the DATA calculation. It should be reiterated here that these steps follow the successful collection of multivariate time series data and the analyses of these data via person-specific factor analysis and dynamic factor modeling. References to “within-time” relate to the former, whereas across time refers to the latter.

#### (1) Raw factor score

The raw factor score is calculated via the following equation:

$$\left[(\%\;CFA\;Variance)\times \frac{({Autoregression}^{2}+\sum {Cross\;Predictions}^{2})}{N_{Factors}}\right]$$ (1)

This calculation returns a score for each factor that is a function of the percent variance in the total symptom variation, accounted for *by that factor* within time and across time. The former is the percent variance from the confirmatory factor model and the latter is the percent variance in the predictive (i.e. time-lagged regression) portion of the dynamic model. Both of these steps are facilitated by utilizing a standardized scale–where all model coefficients are on a |0 to 1| scale. Under these conditions, every factor has a standardized total variance of 1. Thus, for the within-time datum, the percent variance in the overall symptom variation is simply the sum of squared factor loadings for each factor, divided by the number of factors. Meanwhile, the time-lagged regression paths represent the square root of the percent variance predicted in the time-forward variable; for those familiar with bivariate linear regression, this is the relationship between r and r^2^. Thus, the sum of the squared values of *all predictive paths* from a given factor at time (t-1) to all other factors at time (t), divided by the number of factors, will yield the percent of predictive variance in the dynamic model accounted for by each factor. At minimum this will include the autoregression, and can include up to N-1 additional cross-predictions in the dynamic model (where N = number of factors).

#### (2) Normalized factor score

The normalized factor score is calculated via the following equation:

$${FS}_{N}=\frac{Raw\;Factor\;Score}{Max\;Factor\;Score}$$ (2)

This sets the scale for all Factor Scores between 0 and 1, with a fixed maximum of 1.

#### (3) Raw item score

The raw item score is calculated via the following equation:

$$Raw\;Item\;Score=\frac{Item\;Mean}{Max\;Mean}\;x\;\sum \left({FS}_{N}\times |Standardized\;Factor\;Loading\right|)$$ (3)

The item mean reflects the average level of severity (on a 0 to 100 visual analog slider) indicated by the respondent on a given symptom item. Again, dividing by the maximum mean across items normalizes the mean scale between 0 and 1, with a fixed maximum of 1. It seems reasonable to assume that a given item’s relative importance for an individual lies not just in mean levels of that particular item, but also the factor to which that item belongs. If the item belongs to a primary factor, then logically we can conclude it is more important in explaining variation in that individual’s symptom, and thus that item would receive more priority in treatment (vs. items with less explanatory power). Thus, for each item we are generating a score that is a function of (a) the average symptom severity relative to other symptoms; (b) the relative explanatory power–within and across time–for the factor the symptom corresponds to; and (c) the degree to which the symptom relates to the factor.

#### (4) Normalized item score

The normalized item score is calculated via the following equation:

$${IS}_{N}=\frac{Raw\;Item\;Score}{Max\;Item\;Score}$$ (4)

This step normalizes Item Scores between 0 and 1, with a fixed maximum of 1.

#### (5–7) Module score

The module score is calculated via the following equations:

$$Item\text{-}Average\;Module\;Score=\frac{\sum {IS}_{N}}{N_{Items}}$$ (5)

$$Raw\;Sum\;Module\;Score\;=\sum {IS}_{N}$$ (6)

$$Final\;Module\;Score=\frac{Normalized\;(\frac{\sum {IS}_{N}}{N_{Items}})+Normalized\;(\sum {IS}_{N})}{2}$$ (7)

The raw sum preferentially weights those modules with a greater number of items. The assumption here is that a module with a greater number of treatment targets will address a wider range of psychopathology. However, more narrowly defined interventions–such as exposure–might be penalized for being relatively underrepresented in the item-module matching matrix. Thus, the average of the item scores within each module reflects the central tendency of the module, without penalizing modules with fewer items. Taking the average of the (normalized) item-average and raw sum thus provides a balance between a more overtly model-oriented or item-oriented scoring system. The N invoked in the item-average calculation is *not* the total possible number of items in the module. Instead the average is taken over the number of module items present in the model. Items from the total symptom set are often removed during earlier factor analytic steps.

### Selection of modules

**Fig 3:**
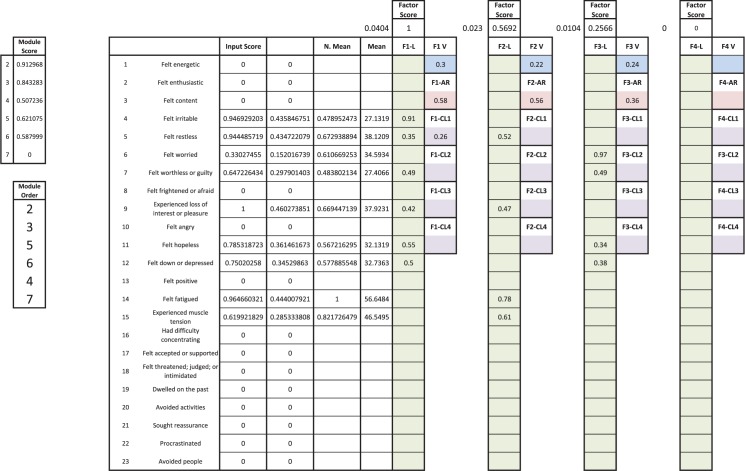
Excel-based calculator for DATA. DATA = Dynamic Assessment Treatment Algorithm.

After the final step has been completed in DATA, the outcome is a module score for each module. These module scores are ordered from greatest to least, with the highest-scoring module being the first administered in treatment, and so on. Module scores range from 0 to 1, and so it is possible that some module scores will have values of 0; in these cases, these modules are not included in the individual’s treatment plan. Returning to the example of participant 021, recall that this individual was provided with modules 2, 3, 4, and 6, but that the treatment team did not select an intervention for anhedonia. However, the input item “experienced loss of interest or pleasure” exhibited a normalized mean of .67 (67% of the highest item mean) and a loading of .42 on the strongest factor (normalized factor score of 1). Thus, not only did DATA select a relevant intervention for anhedonia (Module 5), but it preferentially placed it before Modules 4 and 6. Fig 3 presents the current Microsoft Excel-based DATA interface for participant 021.

## Application of DATA

### Participants

Twenty participants underwent treatment using treatment plans based on expert consensus. Of these 20 participants, most were female (*n* = 16; 80%). The mean age was 30.45 years (SD = 12.4 years). Most participants were White (*n* = 9; 45%); other races reported include Asian/Native Hawaiian/Other Pacific Islander (*n* = 4; 20%), Black/African-American (*n* = 1; 5%), Latino/Hispanic (*n* = 5; 25%), and Other (*n* = 3; 15%). Of the 20 participants, 10 (50%) had a primary diagnosis of GAD, 3 (15%) had a primary diagnosis of MDD, and 7 (35%) had primary diagnoses of both GAD and MDD. Of the participants with a primary diagnosis of GAD, 3 also had a second primary diagnosis of SAD, 1 had a second primary diagnosis of agoraphobia, and 1 had a second primary diagnosis of panic disorder. Of the participants with a primary diagnosis of MDD, one participant had a second primary diagnosis of PTSD.

### Generation of treatment plans

Table 3 provides a side-by-side comparison of the expert consensus-generated treatment plans versus the DATA-generated treatment plans. Points of intersection and divergence are readily apparent in these selections. For instance, both selection methods identified Modules 2 and 3 as the most important interventions for participants 19, 21, and 23, reflecting the primacy of negative emotionality in the participants’ symptom presentation and a corresponding emphasis on tracking and understanding negative emotions. Moreover, for participants 19 and 21, both approaches selected cognitive appraisal and reappraisal for the third module. However, for participant 19, whereas DATA augmented these modules with avoidance interventions (Modules 5 and 7), the expert consensus eschewed any avoidance-based work, instead selecting an intervention for tolerating physical sensations (Module 6). For participant 21, DATA deemphasized the importance of reappraisal strategies, placing this module last and prioritizing Module 5, which explores emotion-driven behaviors, negative reinforcement, and avoidance. Finally, of these three cases, participant 23 demonstrates a significant point of disagreement between DATA and the expert consensus, with the former selecting two additional modules and no overlap in order between the two methods.

**Table 3:** Comparison of treatment team decision model (TTDM) treatment plans with DATA treatment plans.

**ID**	**TTDM**	**DATA**
**1**	2–4–3–5–6	3–2–6–5–4–7
**3**	2–3–4–6	3–2–4–6
**4**	2–7–5–4	5–7–2–3–4–6
**6**	2–3–5–4–7	2–3–4–5–7
**7**	2–3–7–4–5	3–2–5–7–6
**8**	2–5–4–7–6	2–5–3–6–4–7
**9**	2–3–4–5	2–3–5–4–7
**10**	2–3–5–7–4	5–7–6–3–2
**12**	5–2–3–4	7–5–3–2–4–6
**13**	2–3–5–7–4	2–5–7–3–6–4
**14**	2–3–5	2–3–4–6–5–7
**19**	2–3–4–6	2–3–4–5–7
**21**	2–3–4–6	2–3–5–6–4
**23**	2–3–4–5	5–2–4–3–7–6
**25**	2–3–4–5–7	2–5–4–3–7–6
**33**	6–2–3–4	6–2–5–3
**37**	2–3–4–5–7	2–3–4–5
**40**	2–3–4–5–7	3–2–4–7
**48**	4–2–3–5–7–6	6–5–7–3–2–4
68	2-3-4-6-8	2-3-5-4-6-8

*Note*. TTDM = treatment team decision model; DATA = Dynamic Assessment Treatment Algorithm. Numbers refer to modules from the *Unified Protocol for the Transdiagnostic Treatment of Emotional Disorders[25].*

Perhaps the most striking disparity between the two selection processes–and one that seems to underline the influence of human sources of error–is the tendency for the expert panel to select Module 2 as the first module. After the motivational enhancements of Module 1, Module 2 is the formal start to the transdiagnostic CBT interventions provided by the UP. Moreover, the UP is consistent with the canon of manualized treatments for mood and anxiety disorders in its use of psychoeducation to begin therapy. Although purported to be a modular treatment, the UP has an additive structure, in which later modules build upon and reference earlier information and interventions. Thus, for clinicians accustomed to standard applications of CBT, Module 2 fits this additive heuristic, in which psychoeducation serves as a preparatory cornerstone. The algorithm, however, does not have this conceptual handicap and gives no additional weight to earlier modules.

## Discussion

The present study employed a two-stage approach to developing an automated algorithm for targeted, personalized treatment planning. During Stage 1, the study treatment team utilized a set of decision rules to translate person-specific analyses of multivariate time series data into personalized interventions for mood and anxiety psychopathology. After 20 cases were completed, these data were employed to construct an automated algorithm. The results of this effort, DATA, are presented here. DATA is a fully automated algorithm for translating person-specific dynamic assessment data into a targeted, modularized treatment plan. DATA is a powerful empirical tool in that all treatment-planning assumptions and clinical decision points are fully parameterized and digitized, allowing researchers to identify potential points of strength or weakness under controlled conditions. Held in comparison to the decision rules employed by the treatment team during in Stage 1 of the present study, DATA is superior in its ability to quantify the incremental contributions of every input item to the overall pathology and generate a set of item scores that are a function of the mean severity of the item and the predictive strength of the factor (or factors) that the item is associated with.

The ability to quantitate the contribution of each presenting symptom, regardless of semantic, taxonomic, or theoretical assumptions about the symptom’s role, provides two key advantages. First, as we have discussed in regard to the study treatment team, clinicians make treatment decisions based on semantic framing and existing treatment norms. In the present study, this led to the prioritization of symptoms and behaviors most closely related to the semantic labeling of latent factors (and the corresponding devaluation of lesser-loading items). This also led to the tendency to be more likely to place modules in their predetermined order, rather than in an order indicated by the data. In every case of disagreement between the expert consensus and DATA selections, inspection of the DATA output revealed clear quantifications for the divergence (complete DATA results for the 20 cases presented in the current manuscript can be found online at: https://osf.io/xke9b/). That is, the differential ranking of modules was readily traceable to the rank-ordering of item scores, which in turn could be related to item means and factor scores. This feature illustrates the second strength of the DATA approach: All decision rules are quantified. Falsifiability is a central tenet of empirical science. Though DATA appears to be a promising system for building personalized interventions in psychological science, this is clearly an empirical question, and one that could be shown to be incorrect. However, the degree to which this hypothesis may be incorrect, and the specific ways in which it is, should be identifiable and modifiable. The quantitative structure of DATA provides the opportunity to assess the system at multiple levels of analysis and numerical granularity.

An important strength of DATA is its flexibility; more specifically, because the item-module matching matrix is the foundation of DATA, any changes to the three aspects of the item-module matching matrix (items, modules, and relationships between items and modules) can be made and run through the main DATA algorithm. For example, if a clinician specializing in substance use wishes to assess items focusing on substance use (e.g., cravings, use, withdrawal symptoms, etc.), the clinician can substitute the items in the item-module matching matrix with relevant substance use items. If the clinician also wishes to adopt modules more specific to substance use treatment, the modules from the UP can be replaced with the more specified modules. The clinician can then systematically go through each cell of the matrix and determine the degree to which he or she believes a given module targets a given item. The final item-module matching matrix can be run through the DATA algorithm and a similar treatment plan will be outputted. This flexibility is directly related to DATA’s generalizability, in that researchers and clinicians wishing to assess the broad spectrum of psychopathology can modify the matrix as needed while still retaining the calculations delineated above. The algorithm requires only two basic assumptions: that empirically-supported interventions can be identified for each given presenting symptom or behavior, and that each intervention can work in an isolated and modular fashion. That is, the DATA model will not work for interventions with a fixed sequential or additive order.

It is worth noting a couple of limitations of the DATA approach to ensure such limitations can be addressed in future research. First, in its current form, DATA does not specify a cutoff value for module weights. Thus, as long as modules generate a non-zero weight, the module will be assigned to the treatment plan. It is very likely that modules with lower weight values are less relevant to the patient and, if not administered, would not detract from patient outcome in treatment. More work is needed to determine the best way to determine whether a cutoff exists for each person and, if so, how to calculate it. Additionally, the item-module matching matrix was generated via clinician consensus of the current study’s treatment team, but could potentially change if a larger, field-wise consensus were obtained or if clinicians were to opt to create their own matrices based on their interpretation of the symptoms that certain modules target. More research is needed to determine whether a single item-module matching matrix can be used by all clinicians and whether personalization of the item-module matching matrix affects treatment outcome.

DATA evidences great promise for future directions in personalized assessment and treatment of psychological disorders. As research on transdiagnostic factors continues to unfold, the specific inputs used in pre-treatment assessment can be expanded to include a number of potentially relevant symptom domains. Although the current version of DATA matches 13 items to 5 modules, future iterations could include a greater diversity of symptoms and modules without changing the underlying algorithm. As the inputs are refined, the specific cognitive-behavioral modules can also be expanded and refined to better capture the range of symptoms. Such modules can then be tested against other treatment approaches (e.g., standard CBT for depression or anxiety) to determine whether this personalized, modular approach results in similar (if not greater) treatment gains while incorporating time series data and automating the treatment planning process.

## Supporting information

S1 File Phone survey items. (DOCX)
