# Supplementary material for: Development and initial implementation of the Dynamic Assessment Treatment Algorithm (DATA)
Source: PLoS One. 2017 Jun 27;12(6):e0178806. doi: 10.1371/journal.pone.0178806 (PMC5487014; doi:10.1371/journal.pone.0178806)
Supplement: S1 File — (DOCX) [file pone.0178806.s001.docx]

******The phone version of this survey will include a visual analog slider from 0 to 100.**

**To what degree have you:**

1. Felt down or depressed

2. Felt hopeless

3. Felt angry

4. Experienced loss of interest or pleasure

5. Felt frightened or afraid

6. Felt worthless or guilty

7. Felt worried

8. Felt restless

9. Felt irritable

10. Had difficulty concentrating

11. Experienced muscle tension

12. Felt fatigued

13. Felt positive

14. Felt content

15. Felt enthusiastic

16. Felt energetic

**To what degree have you:**

17. Avoided activities

18. Avoided people

19. Procrastinated

20. Sought reassurance

21. Dwelled on the past

22. Felt threatened, judged, or intimidated

23. Felt accepted or supported

**Given Only at the First Measurement Each Morning:**

24. How many hours did you sleep last night?

25. Experienced difficulty falling or staying asleep

26. Experienced restless or unsatisfying sleep?
